# Data from long-term experiments in temperate croplands to evaluate soil organic carbon models

**DOI:** 10.1038/s41597-026-06863-7

**Published:** 2026-02-19

**Authors:** Kenji Fujisaki, Fabien Ferchaud, Hugues Clivot, Elisa Bruni, Bertrand Guenet, Christian Pichot, Antoine Versini, François Baudin, Antonio Bispo, Philippe Peylin, Manuel P. Martin, Johannes L. Jensen, Jørgen Eriksen, Claire Chenu, Andrew S. Gregory, Margaret J. Glendining, Ines Merbach, Nicolas Beaudoin, Bruno Mary, Alain Mollier, Gilles Tison, Christophe Montagnier, Abad Chabbi, Françoise Vertès, Alice Cadéro, Anne-Isabelle Graux, Sylvain Pellerin, Florent Levavasseur, Manon Gilles, Thierry Morvan, Camille Resseguier, Luis Milesi, Alicia Irizar, Adriàn Andriulo, Marie-Noël Mistou, Arnaud Butier, Michel Bertrand, Bénédicte Autret, Marie-Hélène Jeuffroy, Gilles Grandeau, Thierry Doré, Vincent Cellier, Alain Berthier, Sébastien Darras, Guillaume Audebert, Ludovic Pasquier, Fabien Ecalle, Antoine Savoie, Marcus Schiedung, Christopher Poeplau, Nadia I. Maaroufi, Thomas Kätterer, Martin A. Bolinder, Jonathan Sanderman, Pierre Barré

**Affiliations:** 1 INRAE, Info&Sols, 45075 Orléans, France; 2 UMR Eco&Sols, Univ Montpellier, CIRAD, INRAE, IRD, Institut Agro, Montpellier, France; 3 BioEcoAgro Joint Research Unit, INRAE, Université de Liège, Université de Lille, Université de Picardie Jules Verne, 02000 Barenton-Bugny, France; 4 Université de Reims Champagne-Ardenne, INRAE, FARE, Reims, France; 5 Laboratoire de Géologie, École Normale Supérieure, CNRS, PSL Université, IPSL, Paris, France; 6 INRAE, URFM, Avignon, 84000 France; 7 CIRAD, UPR Recyclage et risque, 34000 Montpellier, France; 8 Sorbonne Université, CNRS, ISTeP, 75005 Paris, France; 9 Laboratoire des sciences du climat et de l’environnement (LSCE), IPSL, CEA/CNRS/UVSQ, Gif-sur-Yvette, France; 10 Department of Agroecology, Aarhus University, Blichers Allé 20, Tjele, 8830 Denmark; 11 Université Paris-Saclay, INRAE, AgroParisTech, UMR ECOSYS, 91120 Palaiseau, France; 12 Rothamsted Research, Harpenden, Hertfordshire, AL5 2JQ United Kingdom; 13 Department of Community Ecology, Helmholtz Centre for Environmental Research-UFZ, D-06120 Halle, Germany; 14 INRAE, Bordeaux Sciences Agro, UMR ISPA, 33140 Villenave d’Ornon, France; 15 INRAE, UE APC, Castanet-Tolosan, France; 16 Unité de Recherche Pluridisciplinaire Prairies et Plantes Fourragères, INRAE - Centre de Recherche Nouvelle- Aquitaine-Poitiers, Lusignan, France; 17 INRAE, Institut Agro, UMR SAS, 35000 Rennes, France; 18 INRAE, Institut Agro, UMR PEGASE, 35590 Saint-Gilles, France; 19 INRAE, UEAV, 68000 Colmar, France; 20 INTA, Pergamino, Buenos Aires, Argentina; 21 Université Paris-Saclay, INRAE, AgroParisTech, UMR Agronomie, 91120 Palaiseau, France; 22 INRAE, Unité AgroSystèmes TErritoires Ressources (ASTER), 88500 Mirecourt, France; 23 INRAE, U2E, 21110 Bretenière, France; 24 INRAE, GCIE, 80200 Estrées-Mons, France; 25 INRAE, 11297 UE PAO, 37380 Nouzilly, France; 26 Thünen Institute of Climate-Smart Agriculture, Braunschweig, Germany; 27 Department of Soil and Environment, Swedish University of Agricultural Sciences, Uppsala, Sweden; 28 Department of Ecology, Swedish University of Agricultural Sciences, Uppsala, Sweden; 29 Woodwell Climate Research Center, Falmouth, Massachusetts, USA

**Keywords:** Carbon cycle, Carbon cycle

## Abstract

Soil organic carbon (SOC) models need independent evaluation against field measurements, but those latter are rarely publicly available and harmonized. In this study, we collected and shared data from 167 agronomic treatments in 34 agronomic long-term experiments (LTEs) located in temperate croplands, allowing the evaluation of several soil organic C models such as RothC, Century, AMG, MIMICS, ICBM, Millenial, and CTOOL. The dataset includes climate data, soil properties, C inputs from crops (n = 4588 records) and organic amendments, irrigation data, monthly soil cover, as well as SOC stock measurements in the topsoil layer (n = 1328 records). Climate, soil moisture, and soil temperature data were extracted from daily climate databases. Carbon inputs from crops were calculated from observed yields and harvest index, with some harvest index values estimated, combined with crop allometric coefficients from the literature. Descriptions of LTE, agronomic treatments, methodological metadata, and a part of the code, accompanies the dataset. The dataset can be reused to evaluate single SOC models, or to evaluate an ensemble of models.

## Background & Summary

Soil carbon (C) models are relevant tools to predict C dynamics in terrestrial ecosystems under future global changes. These models need nevertheless independent evaluation and validation using data from controlled long-term experiments (LTEs) with repeated measurements of soil organic C (SOC) stocks^1^. These data are labor expensive and time-consuming to acquire, as SOC changes slowly and soils are heterogeneous. Therefore, initiatives that disseminate and promote the use of data from LTEs for soil C modeling are crucial^2^. However, gathering and harmonizing input data for soil C modeling is a tedious task, and the input datasets supporting modeling research are rarely publicly available^3^. This data paper takes a step forward to overcome such constraints. We collected and estimated input data from 167 agronomic treatments in 34 LTEs, mainly located in France (n = 23 LTEs), where annual crops and temporary grasslands were grown. The data matches required data needed to run and evaluate soil C models such as RothC^4^, Century^5^, AMG^6^, MIMICS^7^, ICBM^8^, Millenial^9^, and CTOOL^10^. The dataset includes climate data, soil properties, C inputs from crops and from organic amendments, irrigation, along with a description of the LTEs and the agronomic treatments. We also collected SOC stock measurements in the topsoil layer (ranging from 0–10 cm to 0–34 cm depth, depending on the site) at several sampling dates in these LTEs, allowing the validation of the SOC changes simulated by the models. The resulting dataset is freely available and reusable.

## Methods

The dataset was conceived according to the following steps. First, we gathered the input variables required to run the C models RothC, Century, AMG, MIMICS, ICBM, Millenial, and CTOOL, as well as the necessary time step associated with each variable. Then, we defined a common template to organize the datasets from the LTEs. We grouped the variables according to their category (climate, soil, C inputs, etc.) and to their required time step. Soil C models often need input variables that are not directly measured in the field and hence need to be estimated from intermediate variables or from independent models. This is for instance the case for the C inputs, which were calculated from crop yields and crop allometric coefficients. The template includes the variables used by the models as well as intermediate variables. The template is included in the dataset (soil_carbon_models_template.xlsx file). The empty tables matching the template were then filled with observed data from the LTEs, depending on data availability. Some variables needed to be estimated, as explained further for each table. Some examples of data retrieving and estimation of variables are also included in the dataset. Part of the data gathered in the dataset were accessible and retrieved from several published datasets: this is the case for the LTEs of Kerbernez^11^, EFELE^12^, Colmar PROspective^13–16^, SIC^17^, Rothamsted Highfield Bare Fallow^18^, Rothamsted Broadbalk^19–23^, Ultuna^24^, Lanna^25^, Lönnstorp^25^, NELITCSE^26^, Waite^27^, and KBS^28,29^.

### Climate data

Climate data were obtained for each LTE using gridded datasets. Average daily and monthly air temperature, daily and monthly precipitations, and monthly reference evapotranspiration (calculated with the Penman-Monteith method^30^ which requires net radiation, air temperature, wind speed, vapor pressure deficit, and atmospheric pressure) were extracted for the periods between the first and last SOC stock measurement. For sites located in mainland France, weather data were extracted from the SAFRAN reanalysis^31^ (source: Météo-France), which delivers gridded daily outputs on a 8 × 8 km grid, from 1958 to the present (https://www.data.gouv.fr/fr/datasets/donnees-changement-climatique-sim-quotidienne, last accessed 11^th^ August 2025). SAFRAN data were downloaded and processed using an Application Programming Interface (API) developed by GéoSAS (https://geosas.fr/web/?page_id = 6345, last accessed 11^th^ August 2025). For sites located in other countries, weather data were extracted and processed from the ERA5^32^ and ERA5-Land^33^ reanalyses. These reanalysis products were extracted using the Open-Meteo API^34^ with the openmeteo package in R^35^. Average daily air temperature was extracted from the ERA5-Land reanalysis, delivered on a 11 × 11 km grid, from 1950 to the present^33^. Daily precipitation was extracted from the ERA5 daily reanalysis, delivered on a 25 × 25 km grid, from 1940 to the present^32^. Reference evapotranspiration available through the Open-Meteo API is calculated with both ERA5 and ERA5-Land data, since some ERA-Land variables are purely interpolated from ERA5 and not a result of the ERA5-Land replay. This is the case for precipitation, short wave radiation, and wind speed, which were internally retrieved from ERA5 by the Open-Meteo API and combined with air temperature retrieved from ERA5-Land to calculate reference evapotranspiration.

Daily soil temperature and soil moisture data were extracted from the ERA5-Land through the Open-Meteo API, with the same method detailed above for climate data. Daily soil temperature and moisture are available for the 0–7 cm and 7–28 cm soil layers; they were weight-averaged to generate data for the 0–28 cm layer.

Climate, soil moisture, and soil temperature data were also extracted before the first measured SOC stock data as it is needed to initialize SOC pool sizes in some models. SAFRAN, ERA5, and ERA5-Land data were extracted with the methodology described above but then averaged over several decades. Mean daily air temperature, daily precipitation, monthly reference evapotranspiration, soil moisture, and soil temperature were calculated for each day and month of the year by averaging all available daily values using data extracted 30 years before the first SOC stock measurement. For some sites, data extraction 30 years before the beginning of the experiment was not possible since SAFRAN and ERA5 data were not available before 1958 and 1940, respectively. For some sites, the period of data extraction was therefore reduced to 16 years. For the oldest LTEs (Folleville, Issoudun, Grignon LTBF, Versailles LTBF), average weather data were calculated using data extracted from SAFRAN from 1959 to 1989. Details of the period considered for the calculation of normal climate data before the beginning of the experiments are provided in the climate_site_metadata table.

For the Rothamsted LTEs site, where the first SOC stock was estimated for 1843, ERA5-Land data are not available in the first decades of the experiment. Therefore, we used the climate data collected at the Rothamsted station (https://www.era.rothamsted.ac.uk/station/rms#datasets), available since the beginning of the experiment. However, only rainfall data were collected at the beginning of the experiment. We thus used the climate data from 1915, where at least wind speed, air temperature, and rainfall were measured. Reference evapotranspiration (Penman-Monteith or FAO-56 method) was calculated using an established method^36^. To match the timeline of SOC stocks and weather data in Rothamsted Broadbalk LTE, SOC stocks data were taken from 1914 to 2010. The climate data for the year 1914, initially missing, was estimated by averaging daily climate data between 1915 and 1945.

### Soil organic carbon stocks

Soil organic carbon (SOC) stocks were computed for each LTE, at each sampling date and for each agronomic treatment. SOC stocks were expressed in Mg ha^−1^ and summed for the topsoil layer, which ranged from 0–10 cm to 0–34 cm depth, depending on the tillage depth. SOC stocks were calculated according to equivalent soil mass principles where possible, in order to take into account possible variation of bulk density over time^37^. The equivalent soil mass correction was mainly done with the SimpleESM R script^38^. For some LTEs, bulk density measurements were not available and hence were estimated using pedo-transfer functions. The methodology used to calculate SOC stocks (bulk density assessment and SOC stocks calculation methods) is provided in the soc table along with SOC stocks values.

### Thermal fractionation

For some LTEs, centennially stable and active fractions of SOC measured by Rock-Eval® thermal analysis^39^ were available (e.g. ref. ^40^) and were therefore gathered in the dataset, as these data can be used to evaluate or initialize the soil C models. The partition between centennially stable and active fractions of SOC was achieved with the PARTY_SOC_V2.0_EU_ model^41^. In the dataset, we reported the stock of the active and centennially stable SOC fraction.

### Physical fractionation

For Lanna, Lönnstorp, Kerbernez, and Versailles LTBF LTEs, physical SOC fractions data were available. We reported in the dataset the relative proportion of organic C (OC) associated with fine fraction (<50 µm; *oc_fine_fraction* in g g^−1^), the stock of OC (*oc_fine_stock* in Mg ha^−1^), and the stock of OC associated with the coarse fraction (>50 µm, *oc_coarse_stock* in Mg ha^−1^). To derive the two fractions for Lanna, Lönnstorp and Kerbernez LTEs, suspended soils were treated with 100 J ml^−1^ of ultrasonication followed by wet sieving to 50 µm to obtain the coarse and fine fraction, as described by Begill *et al*.^42^ and Just *et al*.^43^. For Versailles LTBF LTE, physical SOC fractions data come from Lutfalla *et al*.^44^. Soil samples were fractionated into sand, silt, and clay fractions. The coarse fraction >50 µm was separated by sieving at 50 µm after dispersion of the sand-sized aggregates (overnight shaking with 20 glass beads in 180 mL of deionized water). The silt and clay fractions were then separated by centrifugation after ultrasonic dispersion. The clay and silt fractions were combined in the present dataset in the OC fine fraction.

### Soil properties

Soil properties (particle size distribution, pH, CaCO_3_ content, C:N ratio) were collected for each site in the topsoil layer. Clay content is expressed after sample decarbonatation, as required by the AMG model. pH was measured in water. pH measurement method was not harmonized in the dataset, with soil to water ratio varying from 1:1 to 1:5; this ratio was included in the site_metadata table. C:N ratio was calculated from contents assessed by dry combustion methods. Field capacity and wilting point were only available for a few LTEs (Fagnières LTBF, Bad Lauchstädt LTBF, Ultuna, and NELITCSE). If not available, these variables were estimated with pedotransfer functions. We used Eqs. 1, 2 which were calibrated and validated in French croplands^45^:

1 $${fc}=0.278+0.00245\times {clay}\_{decarb}-0.00315\times {sand}$$

2 $${wp}=0.08+0.00401\times {clay}\_{decarb}-0.000293\times {sand}$$

where *fc* and *wp* are the volumetric soil moisture (cm^3^ cm^−3^) at field capacity (pF = 2.0) and wilting point (pF = 4.2), respectively, with pF denoting the logarithm (base 10) of the soil water tension in cm of water, and *clay_decarb* and *sand* are the clay and sand contents (g 100 g^−1^), respectively. For the PROspective LTE where CaCO_3_ content in soil is about 130 g kg^−1^, *fc* and *wp* were calculated with particle-size fractions measured on samples without decarbonatation^46^.

### Carbon inputs

#### Carbon inputs from crops

The C inputs from crops were calculated using the approach of Clivot *et al*.^47^, which adapted the Bolinder *et al*.^48^ to the French cropping context.

Yields of harvested products from each main crop, cover crop, harvested cover crop, or weeds (if measured), were expressed as Mg dry matter (DM) ha^–1^. Some yield data were occasionally missing and were estimated using averages for the crop in the corresponding treatment. These estimations were sometimes needed to match the timeline of SOC stocks measurements.

Harvest index (HI) of each crop, defined as the fraction of the harvested yield product in the total aboveground biomass was derived from measurements if available (for annual grain crops, measurements are done by cutting the aboveground biomass at ground level at the time of the harvest), or from default values for French crops^47,49^.

Aboveground C inputs (*abg_c_input*) for main crops were calculated from yield and HI, and expressed in Mg C ha^−1^ (Eq. 3):

3 $${abg}\_c\_{input}={yield}\times \frac{\left(1-{HI}\right)}{{HI}}\times {residue}\_{fraction}\times {residue}\_c\_{cont}\times 0.001$$

where *yield* is the yield of the agricultural product for main crops and harvested cover crops (Mg DM ha^−1^), *residue_fraction* is the fraction of non-harvested biomass returned to soil (unitless; between 0 and 1) either measured or based on fixed values per crop, when part of the residues are exported from the field, and r*esidue_c_cont* is the C content of the non-harvested biomass, typically crop residues, either measured or from a default value for French crops (440 g C kg^−1^).

As there is no agricultural product for cover crops and weeds, HI was set to 0 for these crops and therefore not used in the calculation of *abg_c_input* for these crops, and *residue_fraction* was set to 1 (Eq. 4):

4 $${abg}\_c\_{input}={yield}\times {residue}\_{fraction}\times {residue}\_c\_{cont}\times 0.001$$

Belowground C inputs by roots (*root_c_input*) were calculated from crop specific shoot:root ratios, as no measurements of root biomass or rhizodeposition were available. First, the C inputs from root biomass at harvest were calculated as follows and expressed in Mg C ha^−1^ (Eq. 5):

5 $${root}\_c\_{input}=\frac{{yield}}{{shoot}\_{root}\times {HI}}\times {root}\_c\_{cont}\times 0.001$$

where *yield* is the yield of the agricultural product for main crops and harvested cover crops, or the aboveground biomass produced by the cover crops and weeds (Mg DM ha^−1^), *shoot_root* is the shoot:root ratio of the crop (unitless), and *root_c_cont* is the C content of the roots (g C kg^−1^). The shoot:root values were taken from the literature^47,49^. As *root_c_cont* was not available, we used a default value of 400 g C kg^−1^. As there is no agricultural product for cover crops and weeds, HI was set to 0 for these crops and therefore not used in the calculation of root_c_input for these crops (Eq. 6):

6 $${root}\_c\_{input}=\frac{{yield}}{{shoot}\_{root}}\times {root}\_c\_{cont}\times 0.001$$

The extra-root C, produced by root turnover and rhizodeposition (*rhizodep_c_input*), was calculated as follow, according to Bolinder *et al*.^48^ (Eq. 7):

7 $${rhizodep}\_c\_{input}={root}\_c\_{input}\times 0.65$$

The sum of *root_c_input* and *rhizodep_c_input* represents the total belowground C input in the whole soil profile, but it should be scaled to match the sampling depth of SOC stock measurements. This adjustment was done with an asymptotic equation representing the root distribution profile of each crop^50,51^. The belowground C inputs *bg_c_input* was therefore adjusted for sampling depth and calculated as follows and expressed in Mg C ha^−1^ (Eq. 8):

8 $${bg}\_c\_{input}=\left({root}\_c\_{input}+{rhizodep}\_c\_{input}\right)\times (1-{\beta }^{{sampling}{depth}})$$

where *β* is the crop coefficient for root distribution along the whole soil profile, ranging from 0 to 1, and *sampling depth* is the sampling depth used for SOC stock calculation (cm).

Lignin:N ratio of the crop parts (aboveground and root parts), used in the Century model, were taken or estimated from a dataset of chemical quality of crop residues^52^.We associated for each crop a humification coefficient for aboveground carbon inputs that can be used in the the AMG model (table *h_coef_crop.csv* table), taken from a published AMG parameters dataset^49^.

The name of crops in the dataset were harmonized using a naming convention with only subscript letters. When applicable, crops were distinguished between spring-sown and winter-sown crops. When possible, we mapped crop names in the *crop_allom.csv* table to AGROVOC thesaurus^53^ URIs.

#### Carbon inputs from exogenous organic matter applications

Several LTEs in the dataset included applications of exogenous organic matter (EOM) such as manures, slurries, sludges, composts, digestates, etc. C inputs from EOM (*c_eom_input*) were calculated as (Eq. 9):

9 $$c\_{eom}\_{input}={eom}\_{fm}\times {eom}\_{dm}\_{cont}\times {eom}\_c\_{cont}$$

where *eom_fm* is the amount of EOM applied on the field (Mg fresh matter ha^−1^), *eom_dm_cont* is the dry matter content of the fresh EOM product (g kg^−1^), and *eom_c_cont* is the C content of the dried EOM product (g kg^−1^).

Each EOM input was associated with an application date, either recorded (e.g. from published datasets^12,23^) or estimated, using the LTE descriptions found in the literature. Liginin:N ratio of EOM were taken from measured values if available, or estimated from a dataset of C & N mineralization data of several EOMs^54^. Humification coefficients of EOMs for AMG model were taken from measurements if available, or published values^55^. The EOM type names were harmonized by distinguishing the type of product and their origin, such as *bovine manure*, *pig slurry*, *sewage sludge*, etc.

### Monthly soil cover

Monthly soil cover is a parameter used by the RothC model^4^. This parameter was computed for each site and treatment based on the sowing and harvest dates of each crop. For most sites, sowing and harvest dates were available. For some sites, sowing and harvest dates were estimated from usual regional practices. When no crop was growing on the field, the *soil_cover* value was set to 0, whereas when a crop was growing on the field, *soil_cover* value was set to 1.

### Irrigation

Irrigation data were reported at daily time step, as irrigation affects the water balance in the field which has an impact on modelled soil C dynamics.

### Metadata collection

#### Site metadata

Each site of the LTEs was characterized in the table *site_metadata*.csv, using the template developed by Bonares and EJP soil project^56^. The variables in this table include location of the experiment, type of experiment, research themes, institution responsible of the experiment, soil type, land use type, experimental design, and dates of beginning and ending. Some fields in the table were not completed as they were not relevant for soil C modeling, however we added to this table several fields relevant for soil C models: the dominant plant functional type of the experiment; the land use before the beginning of the experiment, and if relevant, the date of land use change. This information is useful to initialize soil C models.

#### Treatments metadata

We characterized each agronomic treatment in the dataset, with basic information regarding soil management practices: mineral nitrogen fertilization (yes/no), organic fertilization (yes/no), irrigation (yes/no), presence of cover crop in the crop rotation (yes/no) and identification of bare fallow treatments. We also characterized the tillage intensity of each agronomic treatment with three classes^57,58^: high-intensity tillage (presence of inversion tillage like ploughing or non-inversion tillage practices at 40 cm depth or below), intermediate intensity tillage (tillage events without inversion and above 40 cm depth), and no-tillage.

## Data Record

The dataset is accessible from a ZIP archive deposited on Recherche Data Gouv^59^. The tables of the dataset are stored in three folders: data, parameters, and metadata. The dataset contains 18 csv tables listed in Table 1. The description of variables in the dataset is provided in the *variables_metadata.csv* table in the metadata folder. A code folder provides R scripts allowing the reproduction of some tables, as detailed in Methods and Code availability sections.

**Table 1:** Description of tables in the dataset.

Table name	Table description	Parameters
climate_data_daily	Daily climate parameters for each site during the experiment	Temperature (°C), precipitation (mm)
climate_data_monthly	Monthly climate parameters for each site during the experiment	Temperature (°C), precipitation (mm), Reference evapotranspiration (mm)
climate_data_daily_spinup	Daily normal temperature and precipitation 16 to 30 years before the beginning of each experiment	Temperature (°C), precipitation (mm)
climate_data_monthly_spinup	Monthly normal temperature and precipitation 16 to 30 years before the beginning of each experiment	Reference evapotranspiration (mm)
soil_moist_temp	Daily soil temperature and moisture in topsoil	Soil temperature (°C), soil moisture (cm^3^ cm^−3^)
soil_moist_temp_spinup	Daily normal soil temperature and moisture in topsoil 16 to 30 years before the beginning of each experiment	Soil temperature (°C), soil moisture (cm^3^ cm^−3^)
soil_properties	Soil properties for each LTE	Particle size distribution, soil pH, CaCO_3_ content (g kg^−1^), C:N ratio, wilting point (cm^3^ cm^−3^), field capacity (cm^3^ cm^−3^)
soc	Measurements of SOC stocks	Sampling date, upper and lower sampling depth (cm), SOC stocks (Mg ha^−1^), thermal fractions, physical fractions
c_inputs_crop	C inputs from crops for each crop cycle in the dataset	Crop name, sowing and harvest dates, yield (Mg ha^−1^), harvest index, C content of crop residues (g kg^−1^), aboveground C inputs (Mg ha^−1^), belowground C inputs (Mg ha^−1^)
crop_allom	Parameters for each crop, used to estimate C inputs or needed by some C models	Crop type, crop name, crop allometry, default harvest index, shoot:root ratio, Beta parameter
h_coef_crop	Humification coefficient for crops	Crop name, AMG humification coefficient
c_inputs_eom	C inputs from each application of exogenous organic matter (EOM)	EOM type, application date, C input, lignin:N ratio, AMG humification coefficient
irrigation	Daily irrigation data for each irrigation event	Date, irrigation amount (mm),
soil_cover	Monthly soil cover during each experiment	Year, month, soil cover (0 or 1)
site_metadata	Metadata of each site	Location, periods of experiment, institution, goal of the LTE, experimental design, type of vegetation, soil type, previous land use
treatments_info	Characteristics of each agronomic treatment	Tillage intensity, bare fallow, organic amendments, mineral nitrogen fertilization, cover crop
climate_site_metadata	Climate data source and method to derive climate data before the beginning of experiment for each site	Site, climate data sources
variables_metadata	Description of dataset variables	Variables names, description, time step, unit

**Fig. 1:**
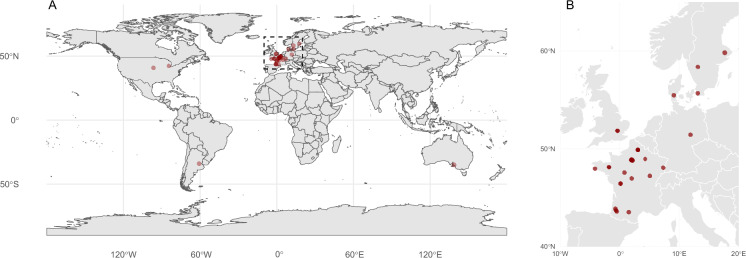
Location of the 34 LTEs globally (A) and in Europe (B).

The dataset gathers data from 34 LTEs, including 167 agronomic treatments. The LTE are described in Table 2 and locations are shown in Fig. 1. Most of the LTE are located in France (n = 23), other LTE come from United Kingdom (n = 2), United States (n = 2), Sweden (n = 3), Denmark (n = 1), Germany (n = 1), Australia (n = 1), and Argentina (n = 1). The duration of data on these LTEs varies from 7 to 96 years. The main research themes of the LTEs are cropping systems experiments (n = 7), bare fallow experiments (n = 7), and mineral fertilization experiments (n = 5). In the 167 agronomic treatments, 37 received EOM amendments, 14 treatments were irrigated, 11 treatments were bare fallows, and 44 treatments included cover crops in the crop rotations. The dataset contains 1328 SOC stock records, and 4588 crop cycle records.

**Table 2:** Long term experiments (LTEs) in the dataset. Lon. and Lat.

LTE	Country	Lon.	Lat.	d	Agronomic treatments	Ref.
auzeville	France	1.51	43.53	35	P fertilization	^70^
doazit	France	−0.63	43.70	12	K fertilization	^71^
folleville	France	1.94	48.84	19	P&K fertilization	^72^
kerbernez	France	−4.13	47.95	27	temporary grassland rotations	^73^
mant	France	−0.50	43.59	17	P fertilization	^74^
tartas	France	−0.73	43.87	21	K fertilization	^74^
colmar_prospective	France	7.33	48.06	18	EOM application and N fert.	^46^
feucherolles_qualiagro	France	1.97	48.90	23	EOM application and N fert.	^75^
le_rheu_efele_pros	France	−1.79	48.11	9	EOM application and N fert.	^76^
le_rheu_efele_tsmo	France	−1.79	48.11	9	EOM application and soil tillage	^76^
lusignan_acbb	France	0.12	46.42	15	duration and fertilization of temporary grasslands	^76^
estrees_mons_sdc	France	3.03	49.87	7	crop rotation and soil tillage	^77^
versailles_la_cage	France	2.13	48.80	22	cropping system	^78^
grignon_sic	France	1.95	48.84	6	cropping system	^79^
issoudun	France	1.99	46.95	21	crop residue management	^80^
estrees_mons_be	France	3.01	49.87	13	bioenergy crops	^81^
pergamino	Argentina	−60.67	−33.85	39	soybean monoculture	^6^
breteniere_res0pest	France	5.10	47.24	10	cropping system	^82^
estrees_mons_res0pest	France	3.03	49.87	10	cropping system	^82^
lusignan_res0pest	France	0.12	46.42	10	cropping system	^82^
nouzilly_res0pest	France	0.79	47.54	10	cropping system	^82^
askov_ltbf	Denmark	9.12	55.47	29	long term bare fallow	^83^
bad_lauchstadt_ltbf	Germany	11.88	51.40	32	long term bare fallow	^84^
grignon_ltbf	France	1.92	48.85	48	long term bare fallow	^83^
rothamsted_ltbf	UK	−0.36	51.80	64	long term bare fallow	^83^
versailles_ltbf	France	2.13	48.80	80	long term bare fallow	^83^
fagnieres_ltbf	France	4.31	48.94	45	long term bare fallow	^85^
ultuna	Sweden	17.65	59.82	63	EOM application and N fert.	^86^
lanna	Sweden	13.13	58.35	28	crop residue management and N fert.	^87^
lonnstorp	Sweden	13.08	55.67	24	crop residue management and N fert.	^87^
broadbalk	UK	−0.37	51.81	95	fertilization and EOM application	^88^
nelitcse	USA	−96.47	40.85	22	tillage and cropping system	^89^
waite	Australia	138.63	−34.97	30	crop rotation	^90^
kbs	USA	−84.40	42.40	10	cropping system	^91^

Columns refer to coordinates of the LTEs; d is the duration in years, between the first and last SOC stock measurement in the dataset – some LTEs may have a larger duration than the duration in the dataset.

## Technical Validation

The dataset was checked by importing all tables in an R environment and running several verification tests. The *check_dataset.R* script reproduces this first technical validation of the dataset. The absence of missing data was checked, as well as the consistency between LTE names and treatments across all tables. The timelines consistency between climate tables, soc table, and C input tables was also confirmed. However, for one LTE (“Cropping systems and soil structure” experiment), the timeline of SOC stocks measurements differed across treatments (n = 28), causing minor mismatch between the different table timelines. Modelers using the dataset should therefore sometimes filter the climate tables to match exactly the SOC stock timeline.

**Fig. 2:**
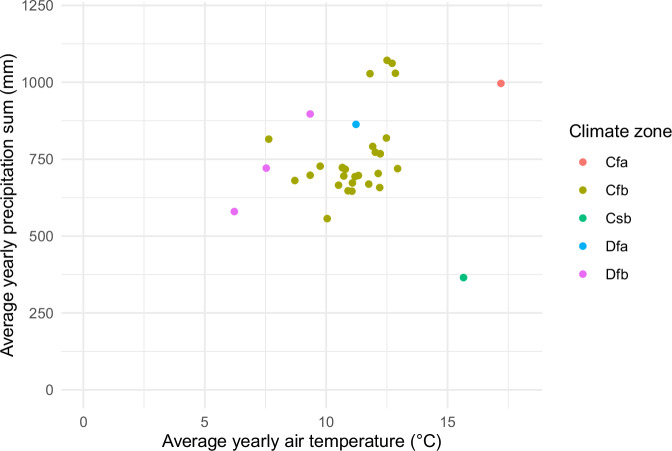
Average yearly climate data and Köppen-Geiger climate zones of the LTEs. Climate zone codes refer to Humid subtropical (Cfa), Temperate oceanic (Cfb), Warm-summer Mediterranean (Csb), Hot-summer humid continental (Dfa), and Warm-summer humid continental (Dfb).

The dataset was also validated by checking data distribution, outliers, and observed trends in SOC stocks and C inputs. The *dataset_desc.RMD* file provided in the code folder of the dataset describes and checks the dataset with a few verification plots from the dataset. The location of each LTE was checked by plotting them on a map (Fig. 1). The physical consistencies of climate forcings from processed climate data were also checked by plotting mean average air temperature and mean average precipitation (Fig. 2). The *check_dataset.R* file also provides code generating climate diagrams at monthly scale for all LTEs.

**Fig. 3:**
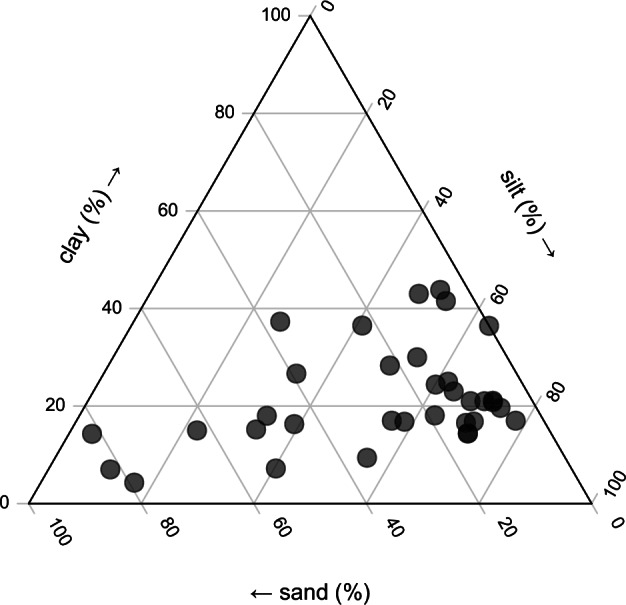
Particle size distribution in the LTEs.

The particle size distribution in the *soil_properties.csv* table was checked and is represented in Fig. 3. Most of the soils have large silt content, with only 6 LTEs having a silt content below 30%.

**Fig. 4:**
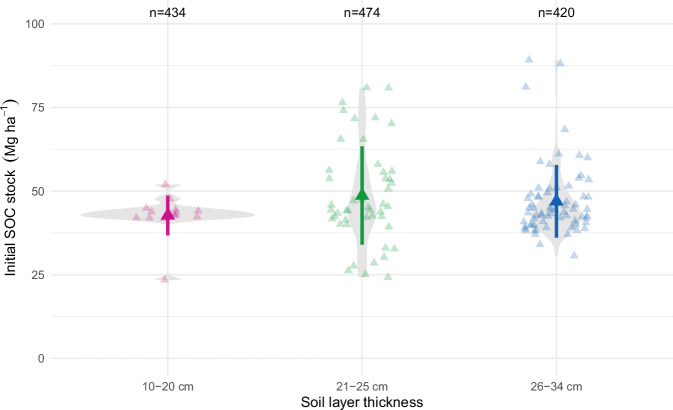
Initial soil organic carbon (SOC) stocks in the LTEs. Each triangle represents an observation for each agronomic treatment in the dataset. Grey area is a smoothed violin plot of SOC stock distribution, including the mean and standard deviation; the width of the violin plot reflects the density of observations.

**Fig. 5:**
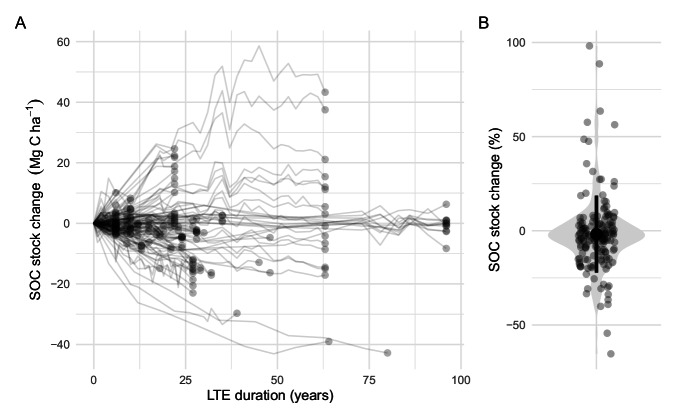
SOC stock changes over time in the LTEs relative to the initial stock (**A**), and distribution of SOC stock changes between last and first measurements (**B**). In panel A, intermediate observations between the first and last SOC stock measurements are masked; lines represent the pure interpolation between observations. In panel B, grey area is a smoothed violin plot of SOC stock changes, including the mean and standard deviation; the width of the violin plot reflects the density of observations.

The SOC stocks distribution and dynamics were checked with several plots. The initial SOC stocks distribution according to soil layer thickness was consistent, with smaller SOC stocks values for the shallowest soil layer thickness (Fig. 4). The SOC stock dynamics over time, shown in Fig. 5A, shows a wide range of variation, from sharp SOC decreases in bare fallow treatments, to large SOC stock increases observed in LTEs where large amounts of EOM were applied. On average, the SOC stock changes between the last and first measurement were slightly negative (Fig. 5B).

**Fig. 6:**
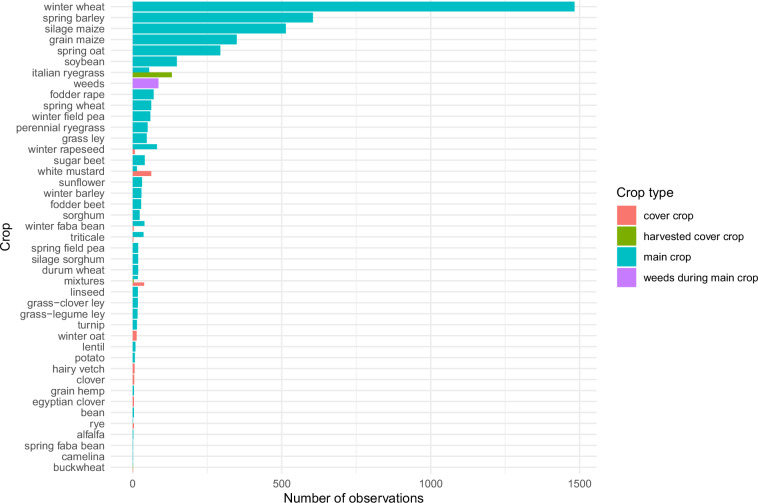
Number of observations per crop and crop type.

**Fig. 7:**
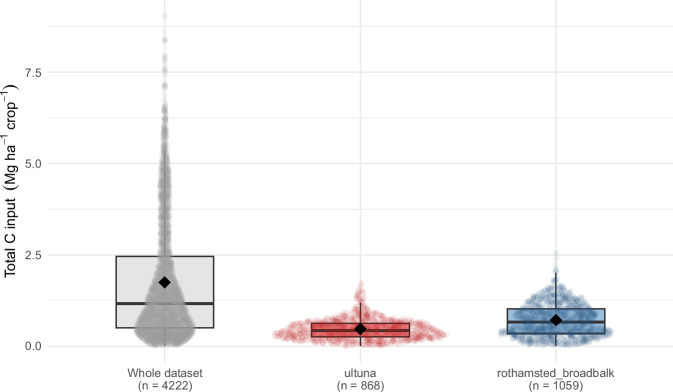
Distribution of total carbon inputs from crops in the whole dataset and in Ultuna and Broadbalk LTEs. Data points are shown as a sina plot; boxplots show medians and interquartile ranges, while diamonds indicate means.

**Fig. 8:**
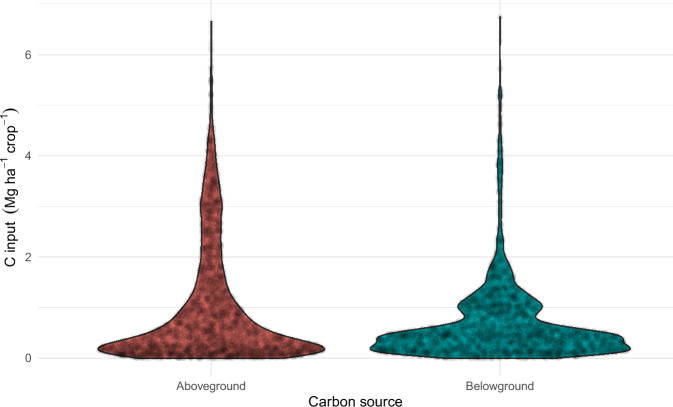
Distribution of aboveground and belowground C inputs from crops.

**Fig. 9:**
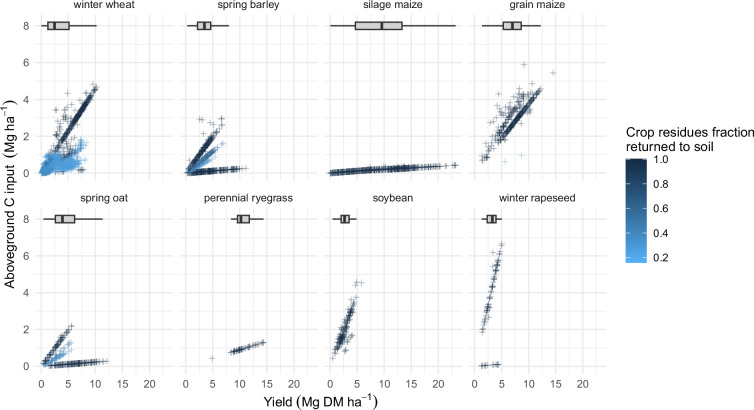
Distribution of yields of the major crops in the dataset, and relationship between aboveground C inputs, yields, and crop residues fraction returned to soil. Each subplot includes a simplified boxplot without outliers of yield distribution.

Data relative to crops and C inputs from crops were also checked with basic tests (absence of NAs, date consistency, absence of duplication). The number of observations per crop and per crop type, shown in Fig. 6, highlights that the most frequently occurring crops in the dataset are winter wheat, spring barley, silage maize, and grain maize. Total C inputs from crops (comprising aboveground and belowground parts) show a wide distribution, with Q1, median, and Q3 values of 0.5, 1.16, 2.45, and a mean of 1.74 Mg C ha^−1^ yr^−1^ (Fig. 7). These C inputs are a slightly lower than current estimates derived from country-scale SOC modeling in croplands in Western Europe, such as 2.14 Mg C ha−¹ yr−¹ reported for France^60^ and 2.7 Mg C ha−¹ yr−¹ for Germany^61^. This discrepancy is largely attributable to the significant weight of the Broadbalk (UK) and Ultuna (Sweden) LTEs in the dataset, which are characterized by low carbon inputs from crops due to limited yields and/or intensive crop residue removal. The distribution of C inputs from aboveground or belowground parts is shown in Fig. 8. The consistency of calculated aboveground C inputs from crops was checked by comparing the aboveground C inputs with crop yields and fraction of crop residues returned to the field (Fig. 9). Overall, the aboveground C inputs are, as expected, smaller when the crop residues are exported from the field, and for crops with a large HI (e.g. silage maize). There are however cases where aboveground C inputs are small even with large crop residue retention (e.g., spring barley, spring oat, and winter rapeseed); these data points derive from the Ultuna LTE, where all crop residues are exported from the field^62^. For this LTE, the yield was considered to be the total aboveground biomass, associated with an estimated HI of 0.95. This effectively results in smaller aboveground C inputs.

A limitation of the dataset is the need to estimate some required variables for SOC models, especially for C inputs. Belowground C inputs are a major source of C flux into the soil, yet they are very rarely measured in LTEs. We relied on estimates of belowground C produced by the crops scaled with the aboveground biomass (shoot:root ratio), i.e., belowground carbon inputs vary with yield. Other studies used fixed estimations of belowground C inputs, yield-independent and not scaled with aboveground biomass variations^63,64^. However, future users may use their own assumptions and methods to estimate C inputs based on measured yields and the HI provided in the dataset. Another limitation of the dataset is that most climatic variables were not directly measured, but came from reanalysis products, which could lead to uncertainties in soil C modeling exercises. Among the models that we identified to define the necessary input variables, only AMG incorporates soil pH as an input variable to calculate the mineralization rate of the active carbon pool^47^. In the present dataset, pH values were not harmonized across different soil-to-water measurement ratios. Previous findings in Polish soils indicate that such differences in ratio resulted in a difference of 0.1 pH units, which was not statistically significant^65^.

The present dataset does not encompass all temperate cropland systems and is heavily biased toward the Western European context. Evaluation of soil carbon models in other temperate regions such as the Americas, Asia, or Eastern Europe using this dataset should therefore be approached with caution. The inclusion of LTE data from these regions would enhance the dataset’s representativeness and reduce geographical bias.

## Usage Notes

The tables of the dataset can be joined by the *site_name* variable, present in all data tables. The link between parameters tables and data tables can be done with the *crop_name* and *crop_type* variables for *crop_allom*.csv and *c_inputs_crop.csv* tables, and with *eom_type* for *h_coef_eom.csv* and *c_inputs_eom.csv* tables. The dataset is provided as a common dataset to run with several soil C models. We did not provide individual input files specifically formatted for each model, as several tools allow to run several models simultaneously^66,67^. Therefore, users who wish to reuse the dataset to evaluate one given model would need to rename and reformat some data to match the input data requirements of the model. Soil layer thickness used in SOC stock calculation varies between sites, from 0–10 cm to 0–34 cm. SOC data were not normalized for a specific soil thickness, as soil thickness normalization may not be required in multi-model simulations in the topsoil layer if model parameters are recalibrated^66^. Users of the dataset dealing with models that require a specific soil thickness (e.g., Century was originally parameterized for 0–20 cm topsoil layer) may need to normalize the SOC stocks and C inputs data.

Users wanting to add new datasets from other temperate LTEs can contact the corresponding author that may decide to update the data paper. In order to facilitate the reuse of these data by placing them in a broader context of interoperability with other data from environmental observations or experiments, their semantic modeling is currently underway. Using methodologies and tools developed by AnaEE France^68^, the variables and their acquisition contexts are modelled based on the generic OBOE ontology^69^ extended to cover the soil carbon domain. This ongoing work will be described in a separate, forthcoming methodology paper, presenting the methodology and semantic resources used and delivering the semantized versions of the data.

## Data Availability

The dataset is accessible from a ZIP archive deposited on Recherche Data Gouv^59^: 10.57745/WKQHW2.

## Code availability

Several R scripts are provided in the code folder of the dataset. Scripts for climate data extraction, aggregation and formatting from SAFRAN (*get_safran_data_geosas_api.R*), ERA5 & ERA5-Land (get_ERA5_data_openmeteo.R), Rothamsted dataset (*climate_data_broadbalk.R*) are provided and can be used to reproduce the climate tables, and soil moisture and temperature tables. The final tables are generated with *merge_climate_data.R*.

The calculation of C inputs from observed data (yields and HI) can be found in the *c_input_calculation.R* file. The *dataset_check.R* script reproduces the checking of the dataset. Description of the dataset including code for the figures used in this paper is found in the *dataset_desc.Rmd* file. The R version used was 4.5.1. The R environment used in this work was encapsulated with the renv package, ensuring code reproducibility within a Rstudio project. This R environment can be restored by running the *setup.R* script.
